# Artificial Intelligence for Intracranial Aneurysm Detection, Rupture-Risk Prediction, and Prognosis Evaluation: Recent Advances and Clinical Integration

**DOI:** 10.3390/diagnostics16182901

**Published:** 2026-09-09

**Authors:** Olti Qirici, Eugen Enesi, Kleona Binjaku, Anduel Kuqi, Indrit Enesi, Ana Ktona, Elinda Kajo Meçe, Arjan Durresi

**Affiliations:** 1 Faculty of Natural Sciences, University of Tirana, 1000 Tirana, Albania; ana.ktona@fshn.edu.al; 2 Service of Radiology, University Hospital Center Mother Theresa, 1001 Tirana, Albania; eugenenesi@gmail.com; 3 Faculty of Information Technology, Polytechnic University of Tirana, 1001 Tirana, Albania; kleona.binjaku@fti.edu.al (K.B.); akuqi@fti.edu.al (A.K.); ienesi@fti.edu.al (I.E.); ekajo@fti.edu.al (E.K.M.); 4 Luddy School of Informatics, Computing, and Engineering, Indiana University, Indianapolis, IN 46202, USA; adurresi@iu.edu

**Keywords:** intracranial aneurysm, rupture risk, prognosis, hemodynamics, radiomics, artificial intelligence

## Abstract

Intracranial aneurysm rupture is a major cause of hemorrhagic stroke and a significant contributor to human mortality. With the emergence of artificial intelligence (AI), particularly machine learning (ML) and deep learning (DL), a series of new approaches have been introduced to improve aneurysm detection, rupture assessment, risk prediction, and prognosis evaluation. This narrative review synthesizes the findings from 21 peer-reviewed studies identified through searches in PubMed, Scopus, Web of Science, and IEEE Xplore, covering the period from June to September 2025. The reviewed studies examined the use of AI for aneurysm detection, rupture assessment and risk prediction, and prognosis evaluation based on clinical, morphological, radiomic, and hemodynamic data. The reviewed studies applied different AI approaches, including conventional ML algorithms, convolutional neural networks, transformer-based models, radiomics, and multimodal models. Several studies reported encouraging performance in aneurysm detection, segmentation, rupture assessment, and prognosis evaluation, while some approaches showed better performance compared with traditional clinical scoring systems. However, important limitations remain, including the frequent use of small or single-center datasets, limited external validation, and heterogeneity in imaging protocols and feature-extraction methods. AI is showing strong potential in supporting the management of intracranial aneurysms. However, further research is needed, particularly through multicenter and external validation, improved model explainability and robustness, and better integration of these systems into clinical workflows.

## 1. Introduction

There are several established clinical scoring systems used in the assessment and management of intracranial aneurysms, including PHASES [1] and UIATS [2]. Additional risk stratification approaches, such as the Glasgow Aneurysm Score [3], have also been proposed in specific clinical settings. However, these methods do not fully account for the dynamic and multifactorial nature of aneurysm rupture risk. In addition, recent developments in ML have led to a surge of AI-powered models that incorporate granular data such as patient history, blood flow simulations, and imaging biomarkers.

These models aim to classify aneurysms as high-risk or stable, thereby supporting clinicians in performing their clinical assessments more effectively.

These techniques have been used not only to assess aneurysm rupture risk but also to analyze clinical symptoms. Following an aneurysm rupture, the aim is to quickly assess and identify the affected area to enable a faster, more efficient intervention. This should increase the probability of timely intervention, thereby averting further damage and improving the patient’s survival rate. Imaging techniques could be utilized, for instance, to perform assessments in a timely manner and determine which intervention technique would best support successful treatment [4].

Surgical intervention does not always occur within the optimal time window, despite the first 72 h being considered the most critical for the intervention [5].

Specific studies have been conducted to show and measure, using AI techniques, the patient’s prognosis following operative treatment (if any).

This paper will review several such articles to better assess the current state of their utilization.

**Figure 1:**
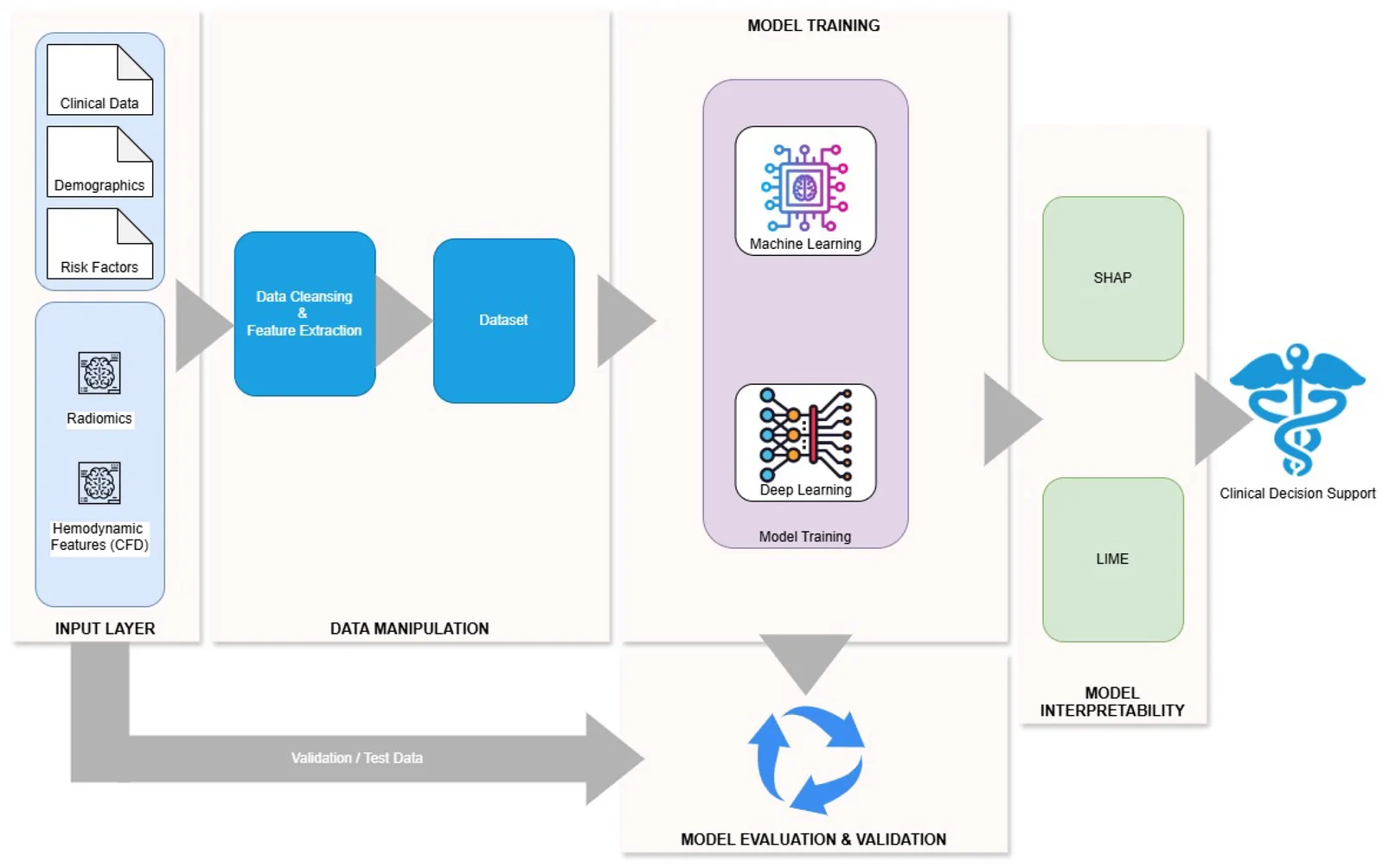
Overview of artificial intelligence workflows for intracranial aneurysm detection, rupture-risk prediction, and prognosis evaluation. Multimodal input data, including clinical information, radiomics, and hemodynamic features derived from computational fluid dynamics (CFD), are processed, starting with data preprocessing and feature extraction to construct datasets for machine learning and deep learning models. The models are evaluated and validated, and interpretability techniques such as SHAP and LIME are applied to support clinical decision-making.

The general workflow for artificial intelligence applications in intracranial aneurysm analysis is illustrated in Figure 1. It includes the entire workflow, from information gathering from data sources to model development and clinical integration.

In this review, the authors have focused primarily on recent publications. They reflect the rapid development of artificial intelligence methods and their increasing application in intracranial aneurysm analysis. The selected literature comprised studies directly relevant to the subject of this paper. A generalized conceptual schema on how these studies have been handled by the original authors is provided in Appendix A, Table A1.

Both original research articles and selected review papers were included to provide a comprehensive and balanced overview of the field. The articles in the review category were used to identify trends and research directions, while greater emphasis was placed on detailed analysis of the original research. For the studies described in the paper, the authors assessed the methodological approaches, data characteristics, and results. In addition, specific limitations have been emphasized and reported.

The remaining parts of this paper are structured as follows: Section 2 covers the relevance of clinical and morphologic variables, with a particular focus on how hypertension, age, aneurysm geometry, and other factors influence rupture and risk models. Section 3 examines the use of artificial intelligence in assessing the risk of intracranial aneurysm rupture, reviewing the key recent literature and methodological trends. Section 4 examines hemodynamic and computational fluid dynamics (CFD)-based machine learning algorithms for predicting treatment results and patient prognosis. Section 5 focuses on deep learning and radiomics frameworks for aneurysm identification and risk prediction. Particular emphasis is placed on imaging-based feature extraction and multimodal integration. The authors have described in Section 6 the main deep learning architectures identified and the principal training methodologies used in the reviewed literature, tracing their evolution from simple CNNs to transformer-based and hybrid models. Section 7 discusses evaluation and validation techniques, focusing on problems such as data heterogeneity, model generalization, and clinical translation. Section 8 and Section 9, respectively, address the explainability of results from AI/ML utilization in intracranial aneurysm and the clinical integration challenges. Finally, Section 10 and Section 11 contain further discussion and comments, followed by Section 12.

## 2. Literature Selection and Review Approach

This study presents a narrative literature review that primarily focuses on recent advances in artificial intelligence applications for intracranial aneurysm detection, rupture-risk prediction, and prognosis assessment. The aim of this review is to provide a structured, up-to-date overview of current methodologies and emerging trends, rather than to conduct a formal systematic review or meta-analysis.

**Figure 2:**
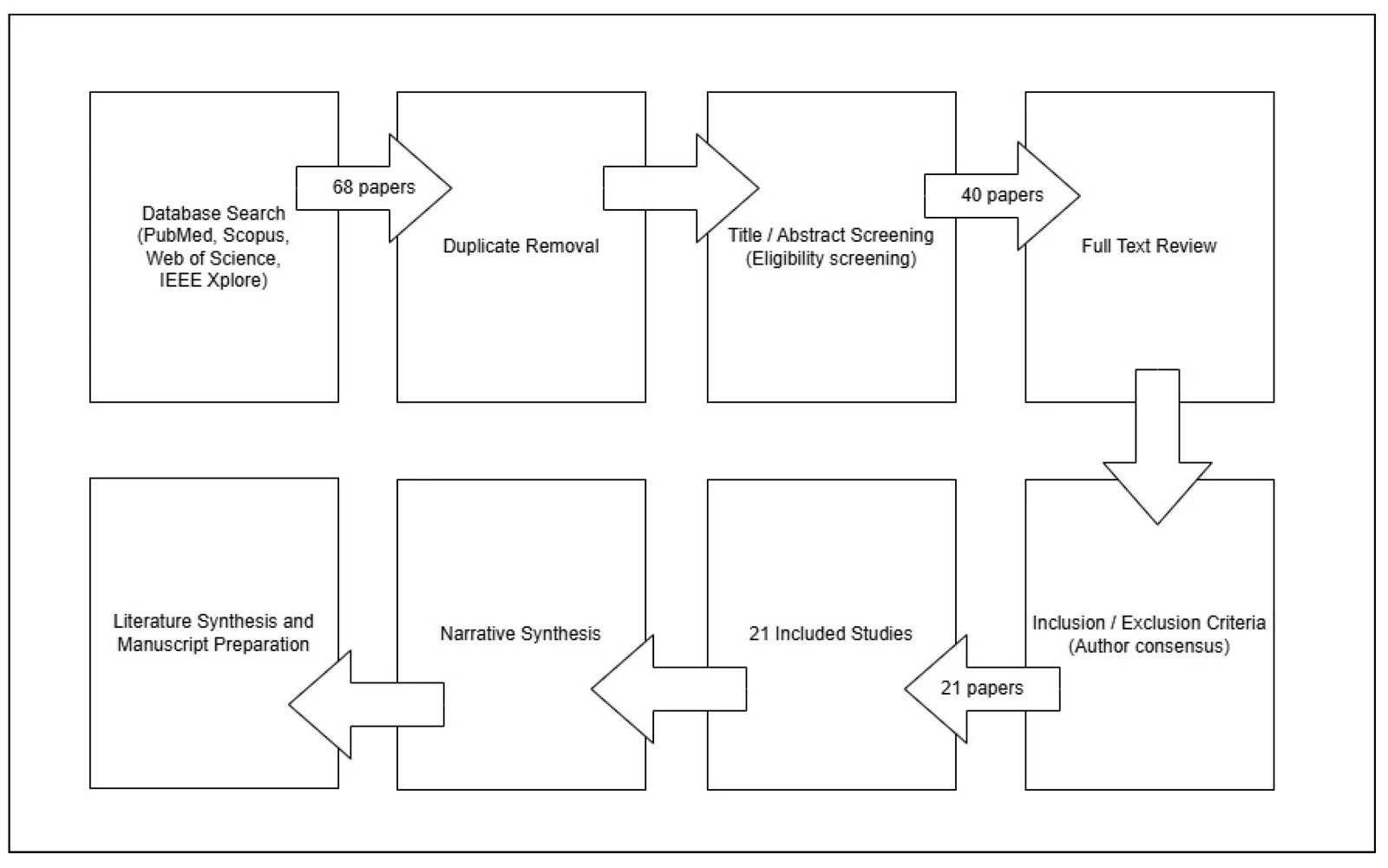
Literature selection and review workflow adopted in this narrative review. The relevant studies were identified through searches of PubMed, Scopus, Web of Science, and IEEE Xplore, resulting in 68 initially identified records. After duplicate removal and title and abstract screening, 40 records proceeded to further assessment. Full-text assessment and application of the predefined inclusion and exclusion criteria resulted in a final set of 21 studies included for qualitative synthesis and comparative analysis.

The selection process was graphically described in Figure 2.

The papers were selected by the authors by searching the major scientific databases, including PubMed, Scopus, Web of Science, and IEEE Xplore. The search for relevant literature was conducted between June and September 2025. It was important to include primarily peer-reviewed studies from the recent period. The publications accepted for review were those published between 2020 and 2025, thus reflecting the recent developments in AI methodologies and their clinical integration. The strategy was based on selecting keyword combinations such as “intracranial aneurysm”, “artificial intelligence”, “machine learning”, “deep learning”, “rupture prediction”, “radiomics”, and “aneurysm detection”. Additional relevant publications were identified through reference tracking of selected articles.

All the titles, abstracts, and conclusions were screened for relevance. The duplicates were removed, and full-text articles were evaluated based on methodological relevance and contribution to the review objectives. The authors collaboratively performed the screening and study selection. The research team, which included contributors with different backgrounds and expertise in computer science, artificial intelligence, medical imaging, and cerebrovascular disease research, thoroughly examined the list of selected papers. Disagreements regarding study relevance were resolved through discussion and consensus. The review primarily includes studies that directly address intracranial aneurysm detection, rupture-risk prediction, prognosis evaluation, segmentation, or AI-assisted imaging analysis. All studies that focused exclusively on non-aneurysmal cerebrovascular conditions (such as ischemic stroke or non-aneurysmal intracerebral hemorrhage), as well as those lacking clear clinical relevance to intracranial aneurysms, were excluded from the corpus of papers.

This narrative approach allows for a comprehensive qualitative synthesis of the literature. It highlights methodological trends, common limitations, and challenges related to these studies (including clinical applicability, data heterogeneity, lack of explainability, and model validation).

Twenty-one studies were ultimately included in the review. These papers present research conducted across multiple countries and regions, reflecting the growing international interest in AI applications in medicine, especially in the analysis of intracranial aneurysms.

During the revision of this manuscript, ChatGPT (OpenAI, GPT-5.6 Sol) was used to assist with language editing and improvement of grammatical clarity and readability. The tool was not used for literature searching, study selection, data extraction, or Scientific analysis.

## 3. Clinical and Morphologic Predictors of Aneurysm Rupture

### 3.1. Clinical Predictors

Clinical characteristics remain fundamental in assessing aneurysm stability and rupture risk. Several factors are consistently associated with increased risk:

- Hypertension: Chronic high blood pressure induces endothelial dysfunction, internal elastic lamina breakdown, and progressive vessel-wall degeneration. It also amplifies hemodynamic stress, making it one of the strongest modifiable risk factors for rupture [1,3].
- Age: Although aneurysm prevalence increases with age, rupture risk exhibits a nonlinear pattern. Younger patients (<50 years) often present with aggressive aneurysm biology, while elderly patients experience rupture due to cumulative vessel-wall fragility and comorbidities [2,3].
- Smoking: Nicotine exposure accelerates extracellular matrix degradation, impairs collagen cross-linking, and enhances chronic inflammation. Studies show smoking roughly doubles rupture risk [1,2].
- Family history and genetic factors: Conditions such as Ehlers–Danlos, ADPKD, and Marfan syndrome predispose individuals to multifocal aneurysms and earlier rupture [1,6].
- Sex: Females, especially post-menopause, carries increased rupture risk due to estrogen-mediated vascular changes [1,2].

### 3.2. Morphologic Predictors

Morphological characteristics derived from CTA, MRA, and DSA provide insight into biomechanical stress and wall integrity.

- Aneurysm Size and Size Ratio (SR): Size > 7 mm increases rupture risk; SR is validated across multiple studies [1,3,6,7].
- Aspect Ratio (AR): AR > 1.6 has been associated with disturbed flow and inflammatory wall degeneration [6,8].
- Irregular Wall Morphology: Blebs, lobulations, and daughter sacs strongly correlate with rupture [1,2,6].
- Location: PComA, AComA, and basilar tip aneurysms display higher rupture risk due to complex bifurcation hemodynamics [1,2,8].
- Wall Shear Stress (WSS): Abnormal WSS states drive endothelial injury or inflammation; AI models integrate WSS with radiomic and morphologic factors [6,7,9,10].
- Previous subarachnoid hemorrhage (SAH): A history of prior aneurysmal rupture is associated with an increased risk of rupture in other existing aneurysms, reflecting underlying vascular vulnerability [1].

These clinical and morphological predictors are also important for AI-based aneurysm assessment, since several are used as input features in ML and DL models. Their combination with radiomic, imaging, and hemodynamic information allows the models to simultaneously evaluate different risk-related characteristics. Therefore, these predictors provide the clinical and morphological basis for the AI-based rupture assessment and risk prediction approaches discussed in the following section.

## 4. Artificial Intelligence for Intracranial Aneurysm Rupture Assessment and Risk Prediction

Several studies have explored the use of machine learning (ML) and deep learning (DL) techniques for intracranial aneurysm rupture assessment and risk prediction. These approaches aim to improve traditional clinical scoring systems by incorporating diverse data sources, including clinical, morphological, radiomic, and hemodynamic features. This section summarizes the main methodologies and discusses their performance and limitations in the context of clinical applicability. Nevertheless, an important distinction should be made between rupture assessment and rupture-risk prediction. Several of the studies reviewed in this section use retrospective datasets containing already ruptured and unruptured aneurysms. Therefore, these models mainly classify rupture status or identify characteristics associated with aneurysm instability rather than directly predicting a future rupture event. A true prediction of future rupture requires a longitudinal follow-up of initially unruptured aneurysms to determine whether they subsequently grow, become unstable, or rupture. This distinction should be considered when interpreting and comparing the performance of the different AI models presented in this section.

A PRISMA-guided systematic review [9] summarized papers published between 2013 and 2023 that examined ML methods for assessing aneurysm rupture and stability. The review aggregated multiple primary datasets—including examples such as 457 unruptured aneurysms in a multi-view CNN, 368 small aneurysms in a CNN-based decision aid, 528 unstable versus 1539 stable cases across ML models, and 1631 cases whose morphology and hemodynamics were analyzed. The models have used methods ranging from classical ML algorithms (SVM, random forest, k-nearest neighbor) to DL approaches (CNNs), followed by radiomics/CFD-augmented frameworks. The reported sensitivities and specificities often exceeded those of conventional clinical scores, indicating that AI methods exhibit promising diagnostic power for instability prediction and, therefore, for predicting the future risk of rupture. Several limitations were emphasized by the authors of this review. Generally, the analyzed studies had relatively small sample sizes. Also, it was reported that there was a lack of multicenter data, insufficient data to select from, and the possibility of spectrum biases. In addition, considerable heterogeneity was reported in imaging and segmentation protocols. There is also an absence of a gold-standard reference, inconsistent external validation, and potential automation bias—all of which affect the generalizability and any probable clinical adoption.

A second PRISMA-based meta-analysis [10] retrieved several aneurysm-related studies published before December 2023 on major databases (MEDLINE, Embase, Cochrane, and Web of Science). From 10,307 records, 20 studies covering more than 20,000 aneurysms were included. The models—ranging from logistic regression, SVM, random forest, and XGBoost to several CNNs—were typically trained using clinical, morphological, radiomic, and CFD hemodynamic features. Although these algorithms achieved strong internal validation and, in many cases, outperformed traditional scores like PHASES and ELAPSS, most lacked external validation. The review noted the high risk of bias, frequent single-center and retrospective designs, inconsistent segmentation protocols, non-uniform outcome definitions (rupture vs. stability), scarce calibration decision-curve analyses, and generally small and convenience cohorts. The authors concluded that while ML offers good discriminative ability, current models are not yet clinically deployable without larger, standardized, multi-institutional validation.

A retrospective study [7] evaluated the TransIAR Net, an end-to-end 3D deep learning model that predicts aneurysm rupture status directly from CTA volumes. The model was trained on 423 patients (449 aneurysms: 141 unruptured, 308 ruptured) from 2009 to 2019 and externally validated on 43 CTA cases across multiple centers. It incorporated dual neighborhood scales (48 and 96 voxels) and a BFS-derived channel focusing on aneurysms and parent arteries, with a transformer block for spatial context and an auxiliary fusion of age, location, and shape. The network outperformed morphology-based baselines, demonstrating that contextual and end-to-end feature learning adds discriminative value. Yet, its retrospective design, class imbalance (few unruptured cases), exclusion of lesions < 3 mm, and small external dataset limit the clinical generalization.

In another investigation, Delucchi et al. [6] used Bayesian networks (BNs) to map interdependencies among rupture-risk factors using data from 1248 patients, 790 of whom had nine phenotypic variables modeled. Both discrete and additive BNs identified the aneurysm size, location, family history, and age at diagnosis as directly linked to the rupture. Meanwhile, several factors like smoking and the presence of hypertension influenced the rupture indirectly by affecting the size and the location (this is true especially for other types of aneurysms). Compared with the regression baselines, BNs provided interpretable probabilistic reasoning, useful for causal inference and clinical decision support. Several limitations were identified, including the single-center design, potential selection bias (under-representation of small or high-risk lesions), and the absence of richer hemodynamic or genetic data.

At a broader level, recent meta-analytic evidence provides a comprehensive overview of machine learning approaches for predicting intracranial aneurysm rupture risk. A systematic review including 36 studies and more than 22,000 patients has evaluated a wide range of models based on 25 different algorithms [11]. Overall, these machine learning techniques demonstrated higher specificity and an improved AUC compared with traditional scoring systems such as PHASES. This was particularly true when imaging and hemodynamic features were incorporated. Despite these encouraging results, the variability in study design and the limited availability of external validation highlight the need for more standardized and prospective investigations.

Beyond the conventional morphological analysis, recent studies have explored the role of hemodynamic information in predicting the risk of intracranial aneurysm rupture. A cohort of 112 patients with known rupture status was studied. Reconstructed time-resolved velocity fields were used to analyze the temporal dynamics of the velocity field using a temporal velocity-informatics framework [12]. By capturing spatial and temporal flow disturbances, machine learning models—particularly support vector machines—achieved strong predictive performance, with an AUC of 0.92 and an accuracy of 86%. These findings suggest that incorporating flow dynamics can enhance rupture-risk assessment, although the approach remains computationally demanding.

However, the predictive performance is not uniform across all aneurysm subgroups. When focusing on small intracranial aneurysms, typically defined as those less than 5 mm, current models appear considerably less reliable [13]. In an analysis of 141 aneurysms, including 101 unruptured and 40 ruptured cases, it was observed that clinical, morphological, and hemodynamic variables did not provide robust predictive power. The results indicate that existing feature sets may not adequately capture the complexity of small aneurysm behavior, underscoring an important limitation of current machine learning approaches.

The prediction of rupture risk becomes even more complex in patients with multiple intracranial aneurysms. In a study of 91 patients with 222 aneurysms, machine learning models were developed to address this challenge using morphological and anatomical features [14]. The resulting model achieved an AUC of 0.843 and an overall accuracy close to 0.80, although the performance was notably better for identifying unruptured aneurysms than ruptured ones. These findings highlight both the potential and current limitations of machine learning in handling clinically complex cases.

Advances in imaging-based frameworks have made it possible to predict automatically the rupture risk directly from angiographic data. In a dataset comprising 335 patients and 500 aneurysms, a fully automated pipeline combining nnU-Net for vessel segmentation and PointNet++ for feature extraction was used to derive aneurysm and vessel shape features from CTA images [15]. These features, together with clinical variables, were used to train multiple machine learning models, with a random forest achieving the best performance (AUC = 0.851, accuracy = 0.782). External validation on an independent dataset further supported the robustness of the approach.

Finally, a multicenter cohort study [16] across eight centers analyzed 1191 CTA scans using the commercial Viz ANEURYSM 3D CNN system, trained on 4351 CTAs to detect unruptured aneurysms ≥ 4 mm. Model predictions were compared with assessments by blinded neuroradiologists. The results demonstrated high sensitivity and substantial time savings, indicating that AI tools can meaningfully augment clinical detection and reduce inter-observer variability, even though full rupture-risk modeling remains a future goal.

Overall, the reviewed studies demonstrate that AI-based models have shown promising results compared with traditional clinical risk scores for rupture assessment and risk prediction, particularly when integrating multimodal data, including clinical, morphological, radiomic, and hemodynamic features.

However, several methodological limitations appear to be common across these studies. As previously emphasized, most data models rely heavily on retrospective data (which is understandable for ethical reasons as well). Based on single-center datasets with limited sample sizes, these often lead to potential overfitting and restricted generalization. Moreover, class imbalances have been identified, particularly the under-representation of unruptured aneurysms. This would further affect the robustness of the analyzed models. Additionally, external validation is often conducted on small cohorts or is entirely absent. A lack of standardized outcome definitions and evaluation protocols was also identified. These factors highlight a gap between high values reported for predictive performance and real-world clinical applicability, underscoring the need for larger, multicenter, and prospectively validated studies.

## 5. Artificial Intelligence for Prognosis and Outcome Prediction in Intracranial Aneurysms

Artificial intelligence approaches have increasingly been applied to patients with intracranial aneurysms to predict clinical outcomes and prognosis. The machine learning models aim to improve the risk stratification and support for personalized decision-making by integrating heterogeneous data sources, including clinical characteristics, imaging findings, and, in some cases, hemodynamic parameters. These approaches are particularly valuable in capturing complex, nonlinear relationships among predictors that are not readily addressed by traditional statistical methods.

In addition to rupture prediction, ML has been applied in emerging studies to assess prognosis and recurrence risk following aneurysm diagnosis or patient treatment. In a retrospective study of 229 patients with posterior circulation aneurysms, ML models were used to identify factors associated with both rupture and recurrence [17]. The analysis revealed that variables such as age, aneurysm size, hypertension, and smoking history were strongly associated with cases of recurrence, while risk for any rupture was linked to the location of the basilar artery, irregular morphologies, and a strong link to familial predisposition. Although the predictive performance was moderate, the study highlights the potential of machine learning to support longitudinal risk assessment and clinical decision-making.

Minardi et al. [18] similarly examined machine learning models—logistic regression, SVM, random forest, and gradient boosting—to forecast results after microsurgical clipping of ruptured AcoA aneurysms. Their retrospective cohort combined clinical, radiological, and intra- and post-operative features such as Fisher grade, aneurysm size and morphology, Hunt–Hess and WFNS grades, and post-operative complications. Outcomes were evaluated using the Glasgow Outcome Scale, dichotomized into good and poor prognosis. The results confirmed that ML can integrate heterogeneous variables to improve personalized outcome assessment following microsurgical treatment.

In another study by Zhang et al. [19], various models—SVM, logistic regression, random forest, and a fully connected neural network (FCNN)—were applied to predict outcomes in patients with aneurysmal subarachnoid hemorrhage (aSAH). Eighteen predictors, including demographic data, aneurysm characteristics, clinical scores, imaging metrics, and blood cell indices, were evaluated in 240 and externally validated in 61 patients. A simple five-variable scorecard identified neutrophil-to-lymphocyte ratio (NLR) as a critical predictor, emphasizing the role of neuroinflammation. The FCNN achieved the best overall performance.

Lei et al. [20] proposed an improved XGBoost-based framework (AFFWDI) combined with an optimized feature-selection algorithm (IPDO) to forecast six-month outcomes after endovascular treatment of intracranial aneurysms. Their multicenter dataset comprised 303 patients from four Chinese hospitals, divided by prognosis according to the Glasgow Outcome Scale. Variables spanned demographics, clinical status, intra-operative parameters, and laboratory results. The AFFWDI model outperformed logistic regression, SVM, and back-propagation neural networks, highlighting the parameters most strongly associated with patient recovery.

In a comprehensive review, Shi et al. [8] summarized the emerging applications of AI in intracranial aneurysm management, from morphological assessment and detection to rupture-risk prediction and outcome forecasting. They reported that while earlier computer-aided detection methods based on region-growing or thresholding offered high sensitivity, they often produced many false positives. More recent ML and deep learning models—particularly logistic regression, random forests, SVMs, and CNNs—have improved diagnostic precision and workflow efficiency, enhancing accuracy and inter-rater reliability.

Finally, Habibi et al. [21] conducted a systematic review and meta-analysis to predict radiological outcomes after aneurysm treatment using ML and deep learning algorithms. The analysis showed that ANNs, SVMs, and ensemble approaches can successfully predict post-treatment occlusion and recurrence, particularly when imaging-derived features are combined with clinical variables. By incorporating multimodal data—clinical, radiological, and hemodynamic—these models achieved greater interpretability and predictive power, underscoring the potential of AI to inform individualized therapeutic planning.

Overall, these studies highlight the growing importance of integrating hemodynamic, clinical, and radiological features in the modeling for aneurysm rupture prognosis. The use of ML models offers an advantage in handling heterogeneous data and capturing nonlinear relationships among predictors. Nevertheless, similar limitations persist. These include, but are not limited to, the reliance on retrospective datasets (as previously stated, also based on ethical reasons), variability in feature selection, and limitations on external validations. Furthermore, the incorporation of CFD-derived features, even though they seem to be physiologically meaningful, introduces an additional variability due to differences in modeling assumptions and simulation protocols. As a result, despite encouraging predictive performance, the translation of these models into routine clinical practice remains limited. Future research should focus on standardizing feature extraction, improving reproducibility, and validating models across diverse patient populations. These findings, including recent studies on recurrence and rupture prediction in specific aneurysm subgroups, further underscore the clinical relevance of machine-learning-based prognostic models.

## 6. Deep Learning and Imaging-Based Approaches for Intracranial Aneurysm Detection

Radiomics represents one of the imaging-based approaches used in aneurysm research by enabling the extraction of high-dimensional quantitative features, such as texture, shape, and intensity, from imaging modalities like CT angiography (CTA) and MR angiography (MRA). These features can reveal subtle image patterns that may escape visual inspection and can subsequently be analyzed using machine learning algorithms. Alongside radiomics, deep learning approaches can directly analyze vascular imaging for aneurysm detection, classification, and segmentation. However, the lack of harmonized imaging standards across different centers continues to limit the reproducibility of results and the transferability of the developed models.

The choice of imaging modality has a substantial influence on the performance and clinical applicability of the AI model. CTA provides a high spatial resolution and is widely used in aneurysm detection and rupture-risk studies. TOF-MRA offers radiation-free vascular imaging, but may be more sensitive to the artifacts related to blood flow. Because of its superior spatial and temporal resolution, DSA remains the reference standard for vascular characterization. However, its invasive nature limits its application in large-scale screening. Consequently, differences in acquisition protocols, scanner variability, and image characteristics contribute to performance variability and reduced generalization across AI studies.

Hu et al. [22] performed a comprehensive multicenter study. They created a deep learning model to detect intracranial aneurysms on CTA scans. The study used a large dataset containing more than 16,000 CTA scans collected from multiple hospitals across China, representing a very high diversity in scanner types and imaging protocols. The evaluation proceeded in three phases: i. image assessment by an experienced radiologist and a comparison of the results with the model’s detection; ii. implementation of the model as a tool that assists the radiologist; iii. validation of the results with real-world clinical data. The model achieved a patient-level sensitivity of 0.957, which outperformed the human readers and significantly reduced the interpretation time. More than 90% of clinicians reported strong confidence in its integration into daily activities, demonstrating that it has been very well generalized across multiple sites and imaging conditions. Although the system was focused on aneurysm detection rather than predicting rupture risk, it demonstrated AI’s potential to serve as a reliable “second reader”, minimizing oversight and standardizing interpretation. The authors emphasized that future work should link this detection framework to morphological, hemodynamic, and clinical data to advance toward personalized modeling of rupture risk. When physicians used the AI system, diagnostic accuracy improved across all groups—most notably for nonradiologists, whose performance approached that of experienced neuroradiologists. This work illustrates how AI can help equalize diagnostic capability, reduce any missed detections, and shorten the reading time. Nevertheless, further validation in clinical outcome studies is required for this system.

The aforementioned ideas were advanced by Ham et al. [23] through a specialized framework for automated aneurysm detection and segmentation using time-of-flight MRA (TOF-MRA). The system employed a skeleton-based 3D patch extraction strategy, combined with semantic segmentation and an auxiliary classification branch, to mitigate the severe imbalance between aneurysm and normal-vessel voxels. The dataset consisted of 154 aneurysm cases for training and 113 external validation cases from the ADAM challenge. It was proposed to modify the 3D U-Net by adding attention and multitask learning capabilities. These changes improved accuracy, achieving 0.91 internal and 0.88 external accuracy. This strong sensitivity was maintained even for detecting aneurysms smaller than 5 mm. The approach improved segmentation stability and reduced false positives, laying the groundwork for quantitative morphology-based rupture-risk prediction.

Hlavata et al. [24] proposed classifying intracranial aneurysms using a CNN with a computationally efficient architecture based on CT scans. They designed a lightweight 2D CNN made up of only three convolutional layers. This design achieved a classification accuracy of 98%, nearly matching the results of deeper models such as ResNet-152. At the same time, the model requires minimal computational power, making it particularly efficient for emergency and low-resource settings. The authors compared their architecture with multiple baselines (ResNet50, ResNet101, and VGG16) and observed similar diagnostic performance at a fraction of the computational cost. However, the model was designed for aneurysm detection, not for evaluating rupture risk. To make it available for predictive applications, it would be necessary to integrate it with morphological and biomechanical indicators.

On these foundations, Paralic et al. [25] built a hybrid 3D CNN–ConvLSTM architecture. Their system was designed to improve the detection of brain aneurysms based on CT angiography and MRI. The model integrates spatial as well as temporal information, providing a richer understanding of vascular morphology and hemodynamic behavior. The network was trained on 33 DSA scans annotated by radiologists and augmented to mitigate the limited sample size (33 samples were insufficient to assess the model’s accuracy). The authors compared the hybrid model against a standard 2D CNN baseline. They found that the 3D CNN–ConvLSTM they built achieved 97.8% accuracy, substantially outperforming the 2D variant in both sensitivity and stability. These results highlight how adding temporal context to spatial modeling enables more consistent differentiation between aneurysmal and normal vessels. Although based on a small dataset, the approach shows the potential for real-time, AI-assisted aneurysm detection once validated on larger cohorts.

Taking a broader view, Gilotra et al. [26] reviewed the recent developments in the use of AI for diagnosing and managing cerebrovascular diseases with a focus on intracranial aneurysms. This synthesis noted that architectures such as U-Net, DeepMedic, and RetinaNet have achieved sensitivities approaching thresholds of 90–100% in retrospective studies. Therefore, they demonstrate that AI can support radiologists in detecting even small or morphologically complex aneurysms. More importantly, this review highlights the transition from detection to prediction of occurrences, particularly by deep learning models that integrate morphological, hemodynamic, and clinical data to estimate rupture probability. Persistent challenges remain, including high false-positive rates, data imbalance, and limited external validation. Yet, the consensus is that AI will increasingly improve patient stratification and enable personalized assessment of cerebrovascular risk.

Overall, deep learning and radiomics-based approaches have significantly advanced the detection and characterization of intracranial aneurysms. Often, this has achieved, or even exceeded, the performance of experienced clinicians who analyzed the same cases. These models benefit immensely from large imaging datasets and sophisticated architectures capable of capturing complex spatial patterns. However, as previously mentioned, key challenges remain, including variability in imaging protocols, a lack of studies harmonizing data from different centers, and limited interpretability of deep learning models. Additionally, many studies focus primarily on detection tasks. To date, fewer efforts have been dedicated to integrating these models into a comprehensive framework for predicting rupture risk. Bridging this gap will require combining detection systems with clinical and hemodynamic data, while also ensuring robust external validation and improving model transparency.

## 7. Deep Learning Architectures

In the reviewed studies for intracranial aneurysm detection, segmentation, and risk assessment described above, the best results were achieved through the support of deep learning architectures. Convolutional neural networks (CNNs) and their derivatives dominate this field. This is especially due to their ability to learn spatial hierarchies directly from medical images. More complex frameworks, including 3D CNNs, U-Nets, residual networks (ResNets), and hybrid models combining CNNs with recurrent or attention mechanisms, extend these capabilities by capturing the volumetric, contextual, and temporal features that are essential for cerebrovascular modeling.

The main architectural patterns and characteristics observed across the reviewed literature are summarized in the following subsections.

### 7.1. Convolutional Architectures

Classical CNNs are widely used to extract morphological and textural cues from angiographic and tomographic data. The lightweight 2D CNNs achieved around 98% accuracy in aneurysm classification while maintaining low computational cost [24]. Deeper residual variants, such as ResNet50–152, provide enhanced generalization but require more data and computational resources. These architectures excel at detecting aneurysms but often require integration with morphological indices (e.g., wall irregularity, aspect ratio, flow velocity) to transition from detection to rupture prediction.

### 7.2. Segmentation-Oriented Frameworks

U-Net-based architectures, widely adopted in the reviewed studies, have become common for accurately delineating aneurysm boundaries [23]. A 3D U-Net with attention and multitask learning addressed class imbalance in TOF-MRA images, achieving an internal accuracy of 0.91 and an external accuracy of 0.88 while preserving sensitivity for small aneurysms (<5 mm) [23]. These designs have enabled a fine-grained wall segmentation which is crucial for quantifying shape irregularity and wall stress—key markers of rupture tendency.

### 7.3. Hybrid and Temporal Models

A 3D CNN–ConvLSTM architecture jointly modeled spatial and temporal information from angiographic scans, achieving 97.8% accuracy and outperforming 2D CNN baselines [25]. Hybrid designs of this kind reflect the shift toward multimodal fusion of hemodynamic and imaging data, bridging the gap between morphology and function in aneurysm analysis.

### 7.4. Attention and Transformer-Based Models

Emerging studies are exploring attention mechanisms and vision transformers (ViTs), which enhance feature localization and contextual awareness. By focusing on salient regions within 3D volumes, these architectures highlight wall defects or irregular curvatures associated with the risk of aneurysm rupture. A 3D Transformer–CNN model outperformed traditional CNNs by modeling long-range dependencies across the vascular network [7]. Such frameworks represent a promising evolution in neurovascular AI, moving from the pure convolution toward learning a global representation.

### 7.5. Training Strategies and Data Augmentation

There is generally limited availability of aneurysm datasets or limited data within existing datasets. To prevent overfitting due to these limitations, training often employs strategies such as cross-modality transfer learning, data augmentation (rotations, intensity variations, synthetic sample generation), or regularization (dropout, batch normalization). It is common to employ hybrid loss functions that combine Dice and cross-entropy metrics for segmentation tasks. The use of multicenter data and synthetic data generation remains important for model generalization and clinical translation.

### 7.6. From Detection to Risk Prediction

While most architectures focus on detecting aneurysms, the next generation of deep learning models is moving toward integrating clinical variables with hemodynamic and radiomic features to produce quantitative estimates of rupture risk. By fusing spatial segmentation with patient-specific parameters, these models enable personalized risk profiling and open the path for clinically explainable AI-based decision support.

## 8. Evaluation and Validation

In the reviewed studies, researchers used a wide range of performance metrics and validation strategies to assess the performance of both traditional and machine learning (ML) models for predicting intracranial aneurysms. The common evaluation indicators included accuracy, F1-score, sensitivity, specificity, precision, and the area under the receiver operating characteristic curve (AUC), along with calibration plots to assess the reliability of predicted probabilities.

To ensure that their findings were robust and generalizable, most teams implemented cross-validation techniques—typically 5-fold or 10-fold designs—and in some cases, both internal and external validation. For instance, recent CTA-based frameworks incorporating automated segmentation and feature extraction have demonstrated consistent performance across both internal and external validation datasets, supporting their robustness in clinical applications [15]. Also, Zhang et al. [19] combined internal and external datasets to assess the consistency of the model’s performance, while Lei et al. [20] evaluated results on separate training and testing sets to avoid overfitting.

The external validation datasets were incorporated into only a limited number of studies. Several such studies reported small reductions in predictive performance, especially when evaluated on other independent datasets. This highlights the persistent challenges encountered in model generalization and robustness across different institutions.

It was observed that the machine learning models frequently demonstrate superior discriminative power when directly compared with established clinical scoring systems. Studies such as that by Minardi et al. [18] found that ML methods were particularly effective at handling complex, nonlinear interactions among variables, especially when incorporating imaging-derived features. The ensemble techniques and the neural networks often achieved not only higher AUC values, but also better calibration. These results suggested more reliable probability estimates than those obtained with conventional tools.

In imaging-centered work—such as that by Shi et al. [8]—ML systems improved detection and segmentation accuracy and worked effectively in conjunction with radiologists, boosting sensitivity and inter-rater agreement. Predictive models for outcomes such as aneurysm rupture, vasospasm, and treatment response often demonstrated encouraging performance compared with traditional regression-based methods.

The clinical implementation of AI systems should be evaluated beyond discrimination metrics such as accuracy and AUC. For the systems to become production-ready, careful evaluation of calibration, threshold selection, and the potential impact of false-positive and false-negative predictions would also be required. In aneurysm management, these considerations are particularly important because both unnecessary interventions and missed rupture risks may carry substantial clinical consequences.

Finally, the meta-analyses and systematic reviews [11,21] have reinforced these trends. It was shown that machine learning models often achieve better predictive performance than standard approaches. Significant sources of inconsistency remain due to variations in dataset quality, feature selection, and design validation. Recent studies have also highlighted reduced model performance in specific subgroups, such as small aneurysms, further emphasizing the challenges of generalizability [13].

## 9. Explainability and Trust

AI-based aneurysm analysis systems are increasingly integrating explainability tools such as SHAP, LIME, and saliency maps because of their importance, especially in medicine. These techniques help to identify which features or image regions contribute most to the model’s predictions. They may also improve the clinician’s confidence in high-stakes decision-making scenarios. A limited number of studies have also explored user-friendly visualization interfaces to facilitate interpretation of AI outputs.

Explainability techniques, despite their usefulness, remain difficult to implement due to their sensitivity to dataset composition, feature correlations, and model architecture. Technical explainability does not always translate into clinically meaningful interpretability. Therefore, visually intuitive explanations may create a risk of overconfidence. The limitations described highlight the need for careful validation and a clinically grounded interpretation of explainable AI systems.

The literature reviewed have also highlighted that model reliability may vary across patient subgroups. Specifically, this is observed as reduced predictive performance for small aneurysms, which can further undermine clinicians’ trust in AI-based decision support systems [13].

A contribution to improving the transparency of model pipelines, making intermediate outputs more accessible for clinical interpretation, has been made possible through end-to-end frameworks that combine automated segmentations with feature extraction from CTA images [15].

The acceptance and adoption of such systems by clinicians can be further enhanced through AI systems that integrate aneurysm detection, segmentation, and classification models through deep learning algorithms. For example, Hu et al. [22] demonstrated that a deep learning model for intracranial aneurysm detection on CT angiography achieved over 90% clinician acceptance across multiple hospitals, indicating that AI can serve as an effective “second reader”. Other studies, including [23,24,25,26], provide additional evidence that combining segmentation, morphological analysis, and hybrid CNN architectures can make AI outputs more interpretable and actionable, ultimately supporting trust and workflow integration.

## 10. Clinical Integration Challenges

AI systems show strong performance in controlled research settings, but significant obstacles limit clinical deployment.

- Imaging Heterogeneity and Diagnostic Variability: Imaging variability across centers affects the reproducibility of radiomic and geometric features. Many AI studies highlight this limitation [4,7,9,16,22,23,24]. More broadly, standardized reporting of imaging datasets, preprocessing, model development, and validation has been recognized as an important requirement for reproducibility in medical imaging AI [27].
- Real-Time Clinical Workflow Constraints: Clinical workflows require rapid, robust PACS-integrated AI tools, especially in emergencies related to time-sensitive intracranial aneurysm [16]. The combination of automated segmentation and feature extraction from CTA images highlights the computational and integration challenges associated with deploying such systems in real-world clinical environments, as identified by a reviewed end-to-end framework [15].
- Uncertainty Quantification and Explainability: Clinicians require interpretable AI and confidence metrics for high-risk treatment decisions. This need is emphasized across the predictive modeling literature [9,10,18,19]. The reviewed studies also highlighted that model performance may degrade in specific subgroups. When dealing with structures such as small aneurysms, additional concerns were raised regarding reliability and clinical trust [13].
- Legal, Ethical, and Liability Issues: AI misclassification raises unresolved medico-legal questions. The literature highlights the lack of mature regulatory frameworks [8,21,26].
- Data Privacy and Regulatory Compliance: Dataset-sharing capabilities remain limited by the privacy constraints of neuroimaging data. Federated learning may help, but it still remains challenging [22,26].
- Need for Prospective Trials: Most AI aneurysm studies are retrospective, but prospective trials are required before real-world clinical adoption [16,22].
  Only a limited number of AI systems for intracranial aneurysm analysis have progressed toward routine clinical deployment or regulatory approval despite the encouraging research performance mentioned throughout this paper. Integration into PACS environments, interoperability with radiology workflows, and compliance with evolving regulatory requirements from bodies such as the FDA and CE remain important barriers to large-scale clinical adoption.

## 11. Discussion

Even though AI applications in intracranial aneurysm management provide considerable performance gains, they still continue to face challenges related to reproducibility, generalizability, and especially in building clinical trust. Interdisciplinary collaboration is essential for refining the models, validating them across multi-institutional datasets, and developing clinically meaningful ways to communicate risk to physicians and patients.

Taken together, the reviewed studies show that the performance of AI models in intracranial aneurysm management depends considerably on the type of data, clinical task, and validation approach. Models developed for detection and segmentation have generally benefited from imaging-based DL architectures, while rupture assessment has involved a broader combination of clinical, morphological, radiomic, and hemodynamic features. Prognosis models have similarly combined clinical and imaging information to estimate patient outcomes. However, the reported performance across these studies cannot always be directly compared because of differences in datasets, sample sizes, imaging modalities, validation methods, and evaluated outcomes. In particular, results obtained from small or single-center retrospective datasets should be interpreted differently from those validated on independent or multicenter cohorts. Another important limitation is class imbalance, particularly in datasets where aneurysm-positive or rupture cases represent a considerably smaller proportion of the available samples. This imbalance can influence model training and reported performance and should therefore be considered when evaluating the reliability and generalizability of the results.

An additional limitation when comparing the reviewed studies is the variability in the reporting of methodological details. Information regarding class distribution, calibration, potential data leakage, and prospective or retrospective study design was not uniformly reported across all studies. Furthermore, performance measures such as AUC, accuracy, and sensitivity were reported for different clinical tasks, datasets, and assessment levels across the reviewed studies and should therefore not be interpreted as directly comparable measures of model performance. Therefore, a direct quality-based ranking of the different models would not be appropriate. These differences should be considered together with dataset size, external validation, and study design when interpreting the reported performance and potential clinical applicability of the models [28].

From a clinical perspective, these differences are important when considering the translation of AI models into routine practice. High performance obtained during model development does not necessarily indicate the same performance across different hospitals, scanners, patient populations, or clinical workflows. Therefore, the clinical value of these models depends not only on their reported accuracy or AUC, but also on external validation, robustness, interpretability, and their ability to support clinical decision-making without disrupting the existing workflow. These considerations are consistent with broader recommendations for transparent and reproducible reporting of clinical AI models [29]. Moreover, the technical or diagnostic performance reported by most of the reviewed studies should not be interpreted as evidence of established clinical effectiveness, since prospective evaluation of workflow impact and clinical outcomes remains limited.

AI-based detection and classification frameworks, such as those described by Hu et al. [22], Ham et al. [23], and Paralic et al. [25], demonstrate that by combining morphological, radiomic, and spatial features, the model interpretation can be improved. This can be achieved by highlighting relevant anatomical structures and lesion characteristics. At the same time, recent end-to-end pipelines that integrate segmentation and feature extraction [15] illustrate how transparency can be enhanced and clinical interpretation facilitated through intermediate model outputs.

To facilitate comparison across the reviewed literature, Table 1 summarizes the principal AI applications by clinical objective, imaging modality, methodological approach, and reported outcomes. The studies were grouped into the categories of detection and segmentation, rupture assessment, risk prediction, and prognosis assessment. The table includes studies reporting directly comparable AI models and clinical applications; therefore, it does not include all 21 studies considered in the broader narrative synthesis.

Nevertheless, the performance variability of these models across datasets and patient subgroups remains a critical concern, as highlighted by the analyzed studies. As mentioned throughout this paper, a reduced predictive rate occurs in specific scenarios, such as those involving small-sized aneurysms [13].

Addressing these limitations is essential for building robust AI systems that can be trusted and safely integrated into clinical workflows.

## 12. Conclusions

This review highlights the growing role of artificial intelligence (AI), machine learning (ML), and deep learning (DL) in intracranial aneurysm detection, rupture-risk prediction, and prognosis assessment. Intracranial aneurysms remain a clinically significant cerebrovascular condition because of their potential to rupture suddenly and cause severe neurological consequences. Recent advances in AI-based imaging analysis and predictive modeling have enabled more detailed evaluation of clinical, morphological, radiological, and hemodynamic factors associated with aneurysm instability and patient outcome. Several studies reviewed in this work demonstrated encouraging results in improving aneurysm detection accuracy, supporting the stratification of rupture risk, and assisting in assessing prognosis through ML and DL methodologies.

The reviewed studies have also demonstrated the rapid evolution of AI architectures and analytical approaches in intracranial aneurysm research. Many systems have been described throughout this paper, including convolutional neural network-based models, transformer-based models, radiomics frameworks, and multimodal predictive systems. All have shown the potential to improve segmentation accuracy, feature extraction, explainability, and individualized risk assessment. At the same time, important limitations remain, including dataset heterogeneity, limited external validation, variability in imaging protocols, and challenges related to clinical integration and interpretability.

The development and validation of AI systems in medicine remain constrained by ethical, regulatory, and data-sharing limitations that often restrict access to large, multicenter datasets. Consequently, many of the studies reviewed in this paper were conducted on relatively limited datasets and under heterogeneous conditions. Nevertheless, as imaging technologies continue to advance and collaborative data-sharing frameworks become more established, mindful of security and ethical concerns, AI-based approaches may enable personalized risk assessment and support more individualized clinical decision-making for patients with intracranial aneurysms.

Several important limitations still remain despite the encouraging results achieved by the reviewed studies. These include data heterogeneity, limited external validation, variability in imaging protocols, and difficulties in integrating AI systems into routine clinical practice. Future studies should focus on multicenter validation, improved model explainability, greater robustness across different populations, and the incorporation of these technologies into existing clinical workflows and decision support systems.

Overall, although substantial challenges remain regarding validation, robustness, and clinical integration, AI-based approaches represent a promising direction for improving intracranial aneurysm detection, rupture-risk prediction, and prognosis assessment. 

## Figures and Tables

**Table 1:** Comparative summary of the studies included in this review, grouped according to their primary clinical objective, namely aneurysm detection and segmentation, rupture assessment, risk prediction, and prognosis assessment. For each study, the table summarizes the principal imaging modality or data source, the applied artificial intelligence methodology, the validation strategy, and the main reported outcome. This comparison highlights methodological trends, differences in validation approaches, and the potential clinical applicability of the reviewed AI systems. The table focuses on studies suitable for direct methodological comparison and therefore represents a subset of the 21 studies included in the narrative review.

Study	Modality	AI Approach	Main Outcome
Detection and Segmentation			
Hu et al. [22]	CTA	Deep CNN	Sensitivity 0.957; outperformed radiologists and reduced interpretation time.
Ham et al. [23]	TOF-MRA	3D U-Net	Robust aneurysm segmentation; external accuracy 0.88.
Hlavata et al. [24]	CT	Lightweight CNN	Classification accuracy of 98% with low computational cost.
Paralic et al. [25]	DSA	3D CNN + ConvLSTM	Detection accuracy of 97.8%; improved spatial–temporal analysis.
Kim et al. [16]	CTA	3D CNN	Improved aneurysm detection and workflow efficiency.
Rupture-Risk Prediction			
Li et al. [7]	CTA + Clinical	Transformer–CNN	AUC 0.92 for rupture-status prediction.
Delucchi et al. [6]	Clinical/Morphologic	Bayesian Network	Identified causal relationships among rupture-risk factors.
Rezaeitaleshmahalleh et al. [12]	CFD	SVM	AUC 0.92 using hemodynamic features.
Feng et al. [14]	Morphologic	ML Models	AUC 0.843 for multiple aneurysm rupture prediction.
Choi et al. [15]	CTA + Clinical	Random Forest	AUC 0.851 with external validation.
Maroufi et al. [11]	Multimodal	Multiple ML/DL	Better predictive performance than PHASES.
Prognosis and Outcome Prediction			
Növer et al. [17]	Clinical + Morphologic	ML Models	Identified factors associated with recurrence and rupture.
Minardi et al. [18]	Clinical + Surgical	LR, SVM, RF	AUC ≈ 0.90 for post-clipping prognosis.
Zhang et al. [19]	Clinical + Imaging	FCNN	NLR identified as a major prognostic factor.
Lei et al. [20]	Clinical + Operative	XGBoost	AUC ≈ 0.93 for outcome prediction.
Habibi et al. [21]	Multimodal	Meta-analysis	Strong performance of ANN- and SVM-based approaches.

## Data Availability

No new data were created or analyzed in this study. Data sharing is not applicable to this article.

## Abbreviations

The following abbreviations are used in this manuscript:

AI	artificial intelligence
ML	machine learning
DL	deep learning
CNN	convolutional neural network
CFD	computational fluid dynamics
CTA	computed tomography angiography
MRA	magnetic resonance angiography
DSA	digital subtraction angiography
SR	size ratio
AR	aspect ratio
PComA	posterior communicating artery
AComA	anterior communicating artery
WSS	wall shear stress
SVM	support vector machine
RF	random forest
k-NN	k-nearest neighbors
XGBoost	extreme gradient boosting
BN	Bayesian network
SHAP	SHapley Additive exPlanations
BFS	breadth-first search
WFNS	World Federation of Neurosurgical Societies scale
aSAH	aneurysmal subarachnoid hemorrhage
NLR	neutrophil-to-lymphocyte ratio
FCNN	fully connected neural network
AFFWDI	adaptive feature fusion with decision integration
IPDO	improved particle dynamic optimization
ANN	artificial neural network
U-Net	U-shaped convolutional neural network
ViT	vision transformer
ConvLSTM	convolutional long short-term memory
AUC	area under the receiver operating characteristic curve
ROC	receiver operating characteristic
AUROC	area under the receiver operating characteristic
PACS	picture archiving and communication system
TOF-MRA	time-of-flight magnetic resonance angiography
UIATS	Unruptured Intracranial Aneurysm Treatment Score
ELAPSS	Earlier subarachnoid hemorrhage, Location, Age, Population, Size, and Shape
PHASES	Population, Hypertension, Age, Size, Earlier subarachnoid hemorrhage, Site
GOS	Glasgow Outcome Scale
ADPKD	autosomal dominant polycystic kidney disease
LASSO	least absolute shrinkage and selection operator
ResNet	residual network

## Appendix A. Summary of Reviewed Studies

Table A1, presents a summary of the reviewed papers. To facilitate comparison across studies, it summarizes datasets, imaging modalities, AI methodologies, validation strategies, and reported performance metrics.

**Table A1:** Detailed characteristics of the literature review including the study objectives, datasets, imaging modalities, artificial intelligence methods, performance metrics, principal findings, and reported limitations. It serves as a detailed reference complementing the comparative synthesis presented in Table 1.

Study	Year	Dataset/Cohort	Modality/Features	AI/ML Method	Main Objective	Performance	Key Findings	Limitations
Xiaopeng et al. [9]	2023	40+ studies	Clinical + Morphologic + CFD	Review/Meta-Analysis	Rupture-risk prediction	—	AI methods outperform PHASES/ELAPSS	Lack of external validation
Daga et al. [10]	2025	20 studies/>20,000 IAs	Clinical + Radiomic + CFD	LogReg, SVM, RF, CNN	Rupture vs. stability prediction	AUC up to 0.92	ML superior to traditional scores	High risk of bias
Li et al. [7]	2023	423 + 43 CTA	Imaging + Clinical	3D Transformer CNN	Rupture-status prediction	AUC 0.92	Contextual features outperform baselines	Small external test, imbalance
Delucchi et al. [6]	2022	1248 patients	Phenotypic variables	Bayesian Network	Risk-factor interplay	—	Identified causal links	Limited features, single center
Kim et al. [16]	2023	1191 CTAs	Imaging (CTA)	3D CNN	Unruptured aneurysm detection	Sens. 0.91	Reduced missed detections	Not full risk modeling
Minardi et al. [18]	2025	AcoA	Clinical + Surgical	LR, SVM, RF	Prognosis after clipping	AUC ≈ 0.9	ML integrates multiple variables	Retrospective, small cohort
Zhang et al. [19]	2023	240 + 61 aSAH	Clinical + Imaging	FCNN	Prognosis prediction	AUC ≈ 0.9	NLR key predictor	Limited validation
Lei et al. [20]	2025	303 (4 centers)	Clinical + Operative	Improved XGBoost (AFFWDI)	Outcome prediction	AUC ≈ 0.93	Identified prognostic features	Complex model, limited generalizability
Shi et al. [8]	2020	—	—	Review	AI in aneurysm management	—	Shift to ML/CNN methods	Early-stage overview
Habibi et al. [21]	2024	36 studies	Clinical + Imaging	Meta-analysis	Post-treatment outcome prediction	—	ANNs and SVMs effective	Retrospective bias
Hu et al. [22]	2024	>16,000 CTA	Imaging	Deep CNN	Aneurysm detection	Sens. 0.957	Outperforms radiologists	No rupture modeling
Ham et al. [23]	2023	154 + 113 TOF-MRA	Imaging	3D U-Net	Aneurysm segmentation	Acc. 0.91/0.88	Robust for small aneurysms	Small dataset
Hlavata et al. [24]	2024	CT	Imaging	2D CNN	Classification	Acc. 98%	Lightweight model	Detection only
Paralic et al. [25]	2023	DSA scans	Imaging	3D CNN + ConvLSTM	Aneurysm detection	Acc. 97.8%	Spatial–temporal insight	Small dataset
Gilotra et al. [26]	2023	—	Review	Review	Cerebrovascular AI	—	Highlights detection → prediction transition	Lack of validation
Maroufi et al. [11]	2025	36 studies/ >22,000 patients	Clinical + Imaging + CFD	Multiple ML/DL models	Rupture-risk prediction	AUC > PHASES	ML outperforms traditional scores	Heterogeneity, limited validation
Rezaeitaleshmahalleh et al. [12]	2025	112 patients	CFD (velocity-based)	SVM, ML models	Rupture-status prediction	AUC 0.92	Hemodynamic features improve prediction	Computational complexity
Swiatek et al. [13]	2025	141 aneurysms	Morphologic + CFD	ML models	Small aneurysm rupture prediction	Low performance	Poor generalizability in small aneurysms	Limited predictive power
Feng et al. [14]	2025	91 patients/ 222 aneurysms	Morphologic + Anatomical	ML models	Multiple aneurysm rupture prediction	AUC 0.843	ML supports complex cases	Class imbalance, small dataset
Choi et al. [15]	2025	335 patients/ 500 aneurysms	CTA + Clinical	RF, ML models	Rupture prediction	AUC 0.851	End-to-end pipeline effective	Sensitive to segmentation quality
Növer et al. [17]	2025	229 patients	Clinical + Morphologic	ML models	Recurrence and rupture prediction	AUC 0.74	Identified key prognostic factors	Moderate performance
